# 2-(2-Meth­oxy­phen­oxy)pyrazine

**DOI:** 10.1107/S1600536811044321

**Published:** 2011-10-29

**Authors:** Shah Bakhtiar Nasir, Zainal Abidin Fairuz, Zanariah Abdullah, Seik Weng Ng, Edward R. T. Tiekink

**Affiliations:** a Department of Chemistry, University of Malaya, 50603 Kuala Lumpur, Malaysia; b Chemistry Department, Faculty of Science, King Abdulaziz University, PO Box 80203 Jeddah, Saudi Arabia

## Abstract

A significant twist is observed in the title molecule, C_11_H_10_N_2_O_2_, as seen in the dihedral angle between the pyrazine and benzene rings of 72.79 (8)°. The meth­oxy group is almost coplanar with the benzene ring to which it is attached [C—O—C—C torsion angle = 175.83 (15)°]. Centrosymmetric dimers are formed in the crystal structure which are held together by weak π–π inter­actions between pyrazine rings [centroid–centroid distance = 3.8534 (10) Å].

## Related literature

For the structure of a related pyrimidine derivative, see: Aznan Akhmad *et al.* (2010 ▶).
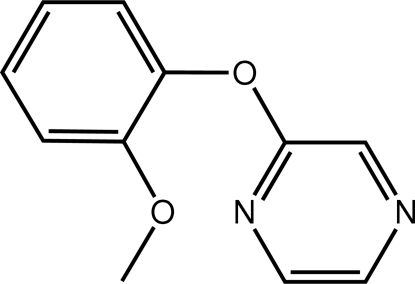


## Experimental

### 

#### Crystal data

C_11_H_10_N_2_O_2_
*M* *_r_* = 202.21
Monoclinic, 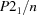

*a* = 7.7497 (10) Å
*b* = 5.8826 (8) Å
*c* = 21.845 (3) Å
β = 92.459 (2)°
*V* = 995.0 (2) Å^3^
*Z* = 4
Mo *K*α radiation
μ = 0.10 mm^−1^
*T* = 293 K
0.35 × 0.3 × 0.2 mm

#### Data collection

Bruker SMART APEX diffractometer
Absorption correction: multi-scan (*SADABS*; Sheldrick, 1996 ▶) *T*_min_ = 0.789, *T*_max_ = 0.862
7364 measured reflections
1743 independent reflections
1262 reflections with *I* > 2σ(*I*)
*R*_int_ = 0.041

#### Refinement

*R*[*F* ^2^ > 2σ(*F* ^2^)] = 0.039
*wR*(*F* ^2^) = 0.107
*S* = 1.03
1743 reflections
138 parameters
H-atom parameters constrained
Δρ_max_ = 0.13 e Å^−3^
Δρ_min_ = −0.13 e Å^−3^

### 

Data collection: *APEX2* (Bruker, 2009 ▶); cell refinement: *SAINT* (Bruker, 2009 ▶); data reduction: *SAINT*; program(s) used to solve structure: *SHELXS97* (Sheldrick, 2008 ▶); program(s) used to refine structure: *SHELXL97* (Sheldrick, 2008 ▶); molecular graphics: *ORTEP-3* (Farrugia, 1997 ▶) and *DIAMOND* (Brandenburg, 2006 ▶); software used to prepare material for publication: *publCIF* (Westrip, 2010 ▶).

## Supplementary Material

Crystal structure: contains datablock(s) global, I. DOI: 10.1107/S1600536811044321/hg5121sup1.cif

Structure factors: contains datablock(s) I. DOI: 10.1107/S1600536811044321/hg5121Isup2.hkl

Supplementary material file. DOI: 10.1107/S1600536811044321/hg5121Isup3.cml

Additional supplementary materials:  crystallographic information; 3D view; checkCIF report

## Figures and Tables

**Fig. 1.**
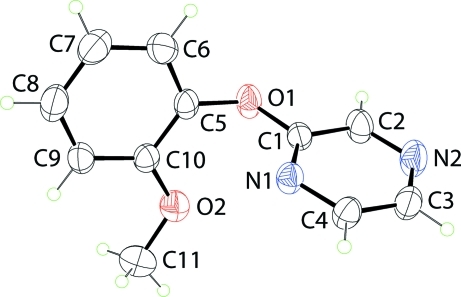
The molecular structure of (I) showing the atom-labelling scheme and displacement ellipsoids at the 35% probability level.

**Fig. 2.**
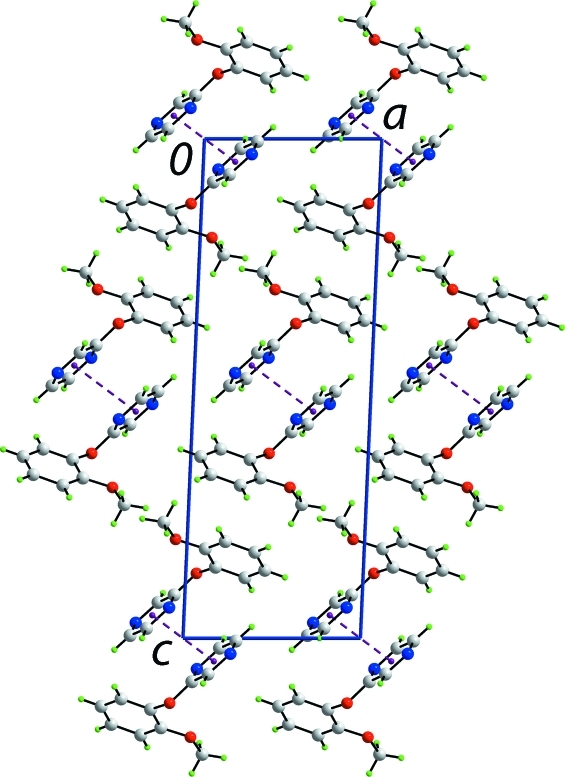
Unit-cell contents for (I) shown in projection down the b axis. The π–π interactions are shown as purple dashed lines.

## supplementary crystallographic information

### Comment

As a part of an on-going synthetic and structural study of *N*-heterocyclic derivatives (Aznan Akhmad *et al.*, 2010), the title compound, (I), was investigated. In (I), Fig. 1, the benzene ring is almost orthogonal to the pyrazine ring, forming a dihedral angle of 72.79 (8)°. The methoxy substituent is co-planar to the benzene ring to which it is connected: the C11—O2—C10—C5 torsion angle is 175.83 (15)°. In the crystal structure, centrosymmetrically related pyrazine rings associate *via* weak π–π interactions [centroid···centroid^i^ distance = 3.8534 (10) Å for *i*: 1 - *x*, -*y*, 1 - *z*]. The dimeric aggregates stack along the *b* axis, Fig. 2.

### Experimental

*o*-Methoxyphenol (2.50 g, 20 mmol) and sodium hydroxide (0.80 g, 20 mmol) were dissolved in water (50 ml) and to the solution was added 2-chloropyrazine (2.28 g, 20 mmol) dissolved in THF (50 ml). The mixture was heated for 6 h. Water was added and the organic phase extracted with chloroform. The chloroform solution was dried over sodium sulfate; slow evaporation led to the formation of colourless crystals.

### Refinement

Hydrogen atoms were placed at calculated positions (C—H 0.93–0.96 Å) and were treated as riding on their parent carbon atoms, with *U*(H) set to 1.2–1.5*U*_eq_(C).

### Crystal data

**Table:**

C_11_H_10_N_2_O_2_	*F*(000) = 424
*M**_r_* = 202.21	*D*_x_ = 1.350 Mg m^−^^3^
Monoclinic, *P*2_1_/*n*	Mo *K*α radiation, λ = 0.71073 Å
Hall symbol: -P 2yn	Cell parameters from 1739 reflections
*a* = 7.7497 (10) Å	θ = 2.8–27.3°
*b* = 5.8826 (8) Å	µ = 0.10 mm^−^^1^
*c* = 21.845 (3) Å	*T* = 293 K
β = 92.459 (2)°	Block, colourless
*V* = 995.0 (2) Å^3^	0.35 × 0.3 × 0.2 mm
*Z* = 4	

### Data collection

**Table:**

Bruker SMART APEX diffractometer	1743 independent reflections
Radiation source: fine-focus sealed tube	1262 reflections with *I* > 2σ(*I*)
graphite	*R*_int_ = 0.041
ω scans	θ_max_ = 25.0°, θ_min_ = 1.9°
Absorption correction: multi-scan (*SADABS*; Sheldrick, 1996)	*h* = −9→8
*T*_min_ = 0.789, *T*_max_ = 0.862	*k* = −6→6
7364 measured reflections	*l* = −25→25

### Refinement

**Table:**

Refinement on *F*^2^	Secondary atom site location: difference Fourier map
Least-squares matrix: full	Hydrogen site location: inferred from neighbouring sites
*R*[*F*^2^ > 2σ(*F*^2^)] = 0.039	H-atom parameters constrained
*wR*(*F*^2^) = 0.107	*w* = 1/[σ^2^(*F*_o_^2^) + (0.051*P*)^2^ + 0.0981*P*] where *P* = (*F*_o_^2^ + 2*F*_c_^2^)/3
*S* = 1.03	(Δ/σ)_max_ = 0.001
1743 reflections	Δρ_max_ = 0.13 e Å^−^^3^
138 parameters	Δρ_min_ = −0.13 e Å^−^^3^
0 restraints	Extinction correction: *SHELXL97* (Sheldrick, 2008), Fc^*^=kFc[1+0.001xFc^2^λ^3^/sin(2θ)]^-1/4^
Primary atom site location: structure-invariant direct methods	Extinction coefficient: 0.105 (7)

### Fractional atomic coordinates and isotropic or equivalent isotropic displacement parameters (Å^2^)

**Table:**

	*x*	*y*	*z*	*U*_iso_*/*U*_eq_	
O1	0.44004 (16)	0.13299 (17)	0.62993 (5)	0.0618 (4)	
O2	0.56589 (16)	0.4818 (2)	0.69804 (6)	0.0658 (4)	
N1	0.58582 (17)	0.3536 (2)	0.56159 (6)	0.0505 (4)	
N2	0.7704 (2)	−0.0324 (2)	0.53106 (8)	0.0716 (5)	
C1	0.5605 (2)	0.1567 (2)	0.58683 (7)	0.0455 (4)	
C2	0.6494 (2)	−0.0375 (3)	0.57169 (8)	0.0626 (5)	
H2	0.6235	−0.1744	0.5905	0.075*	
C3	0.7991 (2)	0.1687 (3)	0.50551 (9)	0.0609 (5)	
H3	0.8842	0.1806	0.4770	0.073*	
C4	0.7080 (2)	0.3568 (3)	0.51984 (8)	0.0535 (5)	
H4	0.7310	0.4928	0.5001	0.064*	
C5	0.3345 (2)	0.3184 (3)	0.64234 (7)	0.0487 (4)	
C6	0.1658 (3)	0.3139 (3)	0.62125 (8)	0.0637 (5)	
H6	0.1254	0.1940	0.5969	0.076*	
C7	0.0555 (2)	0.4879 (4)	0.63627 (9)	0.0711 (6)	
H7	−0.0593	0.4862	0.6219	0.085*	
C8	0.1162 (3)	0.6618 (3)	0.67224 (9)	0.0674 (6)	
H8	0.0420	0.7789	0.6824	0.081*	
C9	0.2854 (2)	0.6670 (3)	0.69370 (8)	0.0571 (5)	
H9	0.3249	0.7873	0.7181	0.068*	
C10	0.3965 (2)	0.4947 (3)	0.67918 (7)	0.0484 (4)	
C11	0.6363 (3)	0.6667 (3)	0.73229 (10)	0.0794 (6)	
H11A	0.7582	0.6441	0.7394	0.119*	
H11B	0.5817	0.6761	0.7708	0.119*	
H11C	0.6166	0.8052	0.7098	0.119*	

### Atomic displacement parameters (Å^2^)

**Table:**

	*U*^11^	*U*^22^	*U*^33^	*U*^12^	*U*^13^	*U*^23^
O1	0.0810 (9)	0.0387 (6)	0.0682 (8)	0.0039 (5)	0.0331 (7)	0.0021 (5)
O2	0.0604 (8)	0.0644 (8)	0.0725 (8)	0.0036 (6)	0.0023 (6)	−0.0089 (6)
N1	0.0558 (9)	0.0438 (8)	0.0529 (8)	0.0063 (6)	0.0124 (7)	0.0032 (6)
N2	0.0788 (12)	0.0533 (9)	0.0851 (11)	0.0142 (8)	0.0294 (9)	−0.0049 (8)
C1	0.0516 (10)	0.0403 (9)	0.0450 (9)	0.0003 (7)	0.0088 (7)	−0.0039 (7)
C2	0.0779 (13)	0.0399 (9)	0.0716 (12)	0.0082 (8)	0.0209 (10)	0.0004 (8)
C3	0.0575 (11)	0.0613 (11)	0.0655 (11)	0.0062 (9)	0.0200 (9)	−0.0036 (9)
C4	0.0531 (10)	0.0532 (10)	0.0551 (10)	0.0039 (8)	0.0130 (8)	0.0054 (8)
C5	0.0584 (11)	0.0415 (9)	0.0479 (9)	0.0004 (8)	0.0209 (8)	0.0017 (7)
C6	0.0681 (13)	0.0671 (12)	0.0569 (11)	−0.0133 (10)	0.0153 (9)	−0.0117 (9)
C7	0.0531 (12)	0.0906 (15)	0.0703 (12)	0.0018 (10)	0.0112 (9)	−0.0025 (12)
C8	0.0642 (13)	0.0642 (12)	0.0758 (13)	0.0118 (10)	0.0244 (10)	−0.0003 (10)
C9	0.0637 (12)	0.0478 (10)	0.0612 (11)	−0.0007 (8)	0.0204 (9)	−0.0056 (8)
C10	0.0539 (10)	0.0449 (9)	0.0476 (9)	−0.0006 (8)	0.0153 (7)	0.0024 (7)
C11	0.0816 (15)	0.0739 (14)	0.0819 (14)	−0.0131 (11)	−0.0062 (11)	−0.0045 (11)

### Geometric parameters (Å, °)

**Table:**

O1—C1	1.3611 (18)	C5—C6	1.368 (2)
O1—C5	1.3969 (18)	C5—C10	1.386 (2)
O2—C10	1.361 (2)	C6—C7	1.382 (3)
O2—C11	1.416 (2)	C6—H6	0.9300
N1—C1	1.3013 (19)	C7—C8	1.362 (3)
N1—C4	1.343 (2)	C7—H7	0.9300
N2—C2	1.319 (2)	C8—C9	1.374 (3)
N2—C3	1.331 (2)	C8—H8	0.9300
C1—C2	1.382 (2)	C9—C10	1.376 (2)
C2—H2	0.9300	C9—H9	0.9300
C3—C4	1.356 (2)	C11—H11A	0.9600
C3—H3	0.9300	C11—H11B	0.9600
C4—H4	0.9300	C11—H11C	0.9600
			
C1—O1—C5	118.61 (11)	C5—C6—H6	120.1
C10—O2—C11	117.50 (14)	C7—C6—H6	120.1
C1—N1—C4	115.09 (13)	C8—C7—C6	119.45 (19)
C2—N2—C3	116.06 (14)	C8—C7—H7	120.3
N1—C1—O1	120.31 (13)	C6—C7—H7	120.3
N1—C1—C2	123.27 (14)	C7—C8—C9	120.97 (17)
O1—C1—C2	116.42 (14)	C7—C8—H8	119.5
N2—C2—C1	121.24 (16)	C9—C8—H8	119.5
N2—C2—H2	119.4	C8—C9—C10	120.18 (17)
C1—C2—H2	119.4	C8—C9—H9	119.9
N2—C3—C4	122.03 (16)	C10—C9—H9	119.9
N2—C3—H3	119.0	O2—C10—C9	125.19 (16)
C4—C3—H3	119.0	O2—C10—C5	116.11 (14)
N1—C4—C3	122.29 (15)	C9—C10—C5	118.71 (17)
N1—C4—H4	118.9	O2—C11—H11A	109.5
C3—C4—H4	118.9	O2—C11—H11B	109.5
C6—C5—C10	120.86 (15)	H11A—C11—H11B	109.5
C6—C5—O1	118.58 (15)	O2—C11—H11C	109.5
C10—C5—O1	120.38 (16)	H11A—C11—H11C	109.5
C5—C6—C7	119.83 (17)	H11B—C11—H11C	109.5
			
C4—N1—C1—O1	179.84 (14)	O1—C5—C6—C7	175.72 (14)
C4—N1—C1—C2	−1.0 (2)	C5—C6—C7—C8	−0.3 (3)
C5—O1—C1—N1	5.6 (2)	C6—C7—C8—C9	0.1 (3)
C5—O1—C1—C2	−173.59 (15)	C7—C8—C9—C10	−0.2 (3)
C3—N2—C2—C1	−0.7 (3)	C11—O2—C10—C9	−4.0 (2)
N1—C1—C2—N2	1.7 (3)	C11—O2—C10—C5	175.83 (15)
O1—C1—C2—N2	−179.10 (16)	C8—C9—C10—O2	−179.69 (16)
C2—N2—C3—C4	−0.8 (3)	C8—C9—C10—C5	0.5 (2)
C1—N1—C4—C3	−0.5 (3)	C6—C5—C10—O2	179.48 (14)
N2—C3—C4—N1	1.5 (3)	O1—C5—C10—O2	4.4 (2)
C1—O1—C5—C6	106.12 (17)	C6—C5—C10—C9	−0.7 (2)
C1—O1—C5—C10	−78.74 (19)	O1—C5—C10—C9	−175.73 (13)
C10—C5—C6—C7	0.6 (3)		
